# Crystal structure and Hirshfeld surface analysis of a pyridiniminium bromide salt: 1-[2-([1,1′-biphen­yl]-4-yl)-2-oxoeth­yl]-3-methyl-1,4-di­hydro­pyridin-4-iminium bromide

**DOI:** 10.1107/S2056989018006217

**Published:** 2018-04-27

**Authors:** S. N. Sheshadri, Huey Chong Kwong, C. S. Chidan Kumar, Ching Kheng Quah, B. P. Siddaraju, M. K. Veeraiah, Muhammad Aiman Bin Abd Hamid, Ismail Warad

**Affiliations:** a Department of Chemistry, GSSS Institute of Engineering & Technology for Women, Mysuru 570 016, Karnataka, India; b School of Chemical Sciences, Universiti Sains Malaysia, Penang 11800 USM, Malaysia; c Department of Engineering Chemistry, Vidya Vikas Institute of Engineering & Technology, Visvesvaraya Technological University, Alanahally, Mysuru 570 028, Karnataka, India; d X-ray Crystallography Unit, School of Physics, Universiti Sains Malaysia, 11800 USM, Penang, Malaysia; e Department of Chemistry, Cauvery Institute of Technology, Mandya 571 402, Karnataka, India; f Department of Chemistry, Sri Siddhartha Institute of Technology, Tumkur 572 105, Karnataka, India; g Department of Chemistry, Science College, An-Najah National University, PO Box 7, Nablus, West Bank, Palestinian Territories

**Keywords:** crystal structure, ionic liquids, pyridiniminium salt, hydrogen bonding, Hirshfeld surface analysis

## Abstract

In the cation of the title salt, C_20_H_19_N_2_O^+^·Br^−^, the phenyl rings are inclined to one another by 38.38 (8)°, whereas the central phenyl ring and the pyridiniminium ring are almost perpendicular with a dihedral angle of 87.37 (9)°. The N^+^=C cationic double bond was verified by the shortened bond length of 1.337 (2) Å. In the crystal, the Br^−^ anion is linked to the cation by an N—H⋯Br hydrogen bond. C—H⋯O hydrogen bonds link adjacent pyridiniminium cations into inversion dimers with an *R*_2_^2^(18) graph-set motif. These dimers are stacked in a phen­yl–phenyl T-shaped geometry through C—H⋯π inter­actions. A Hirshfeld surface analysis was conducted to verify the contributions of the different inter­molecular inter­actions.

## Abstract 2

The Br^−^ anion is linked to the cation by an N—H⋯Br hydrogen bond. C—H⋯O hydrogen bonds link adjacent pyridiniminium cations into inversion dimers with an 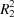
(18) graph-set motif. These dimers are stacked in a phen­yl–phenyl T-shaped geometry through C—H⋯π inter­actions.

## Chemical context   

Over the past decade, ionic liquids have been the subject of intense research as a customizable replacement for volatile organic solvents because of their negligible vapor pressure, excellent thermal stability, high ionic conductivity and solvation ability (Davis, 2004 ▸). A wide range of applications using ionic liquids has been reported in many areas, such as their use as homogeneous and heterogeneous catalysts (Dong *et al.*, 2016 ▸) and biological reaction media (Lopes *et al.*, 2017 ▸), and in nuclear waste treatment (Ha *et al.*, 2010 ▸) and water purification (Fuerhacker *et al.*, 2012 ▸; Wang & Wei, 2017 ▸).

In the view of the above and of our research inter­est in the synthesis of ionic liquids, we present in this study the crystal structure and Hirshfeld surface analysis of the title pyridin­iminium halide salt.
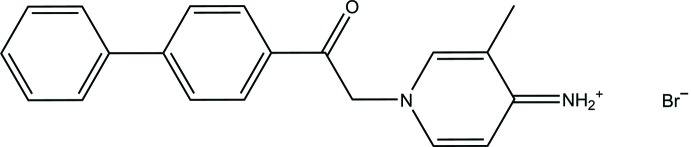


## Structural commentary   

**Figure 1:**
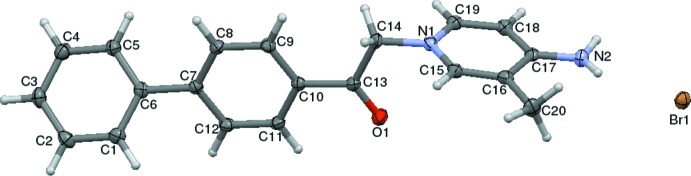
The mol­ecular structure of the component ions of the title salt, indicating the atom-numbering scheme. Displacement ellipsoids are drawn at the 50% probability level.

Fig. 1 ▸ shows the asymmetric unit of the title salt, consisting of one 1-(2-([1,1′-biphen­yl]-4-yl)-2-oxoeth­yl)-3-methyl­pyridin-4(1*H*)-iminium cation and one bromide anion. The cation is constructed from a pyridiniminium ring (N1/C15–C19) and a biphenyl unit (C1–C6 and C7–C12), inter­connected by a (C=O)—C ketone bridge. The biphenyl conformation experiences non-bounded steric repulsion between *ortho*-hydrogen atoms (Poater *et al.*, 2006 ▸), with the phenyl rings inclined to one another by 38.38 (8)°. The second phenyl ring (C7–C12) is nearly parallel to the ketone bridge (O1/C13–C14), as shown by the torsion angles C9—C10—C13—O1 [−179.10 (18)°] and C9—C10—C13—C14 [1.7 (2)°]. Conversely, this phenyl ring is almost perpendicular to the pyridin­iminium ring [dihedral angle = 87.37 (9)°]. The bond lengths and angles in the cation are generally within normal ranges. However, the N2—C17 bond [1.337 (2) Å] is shorter than expected for an NH_2_—C_ar_ single bond [1.38 (3) Å] although similar bond lengths have been observed in related compounds with an N^+^=C double bond (Chidan Kumar *et al.*, 2017 ▸; Sharmila *et al.*, 2014 ▸; Yue *et al.*, 2013 ▸).

## Supra­molecular features   

**Figure 2:**
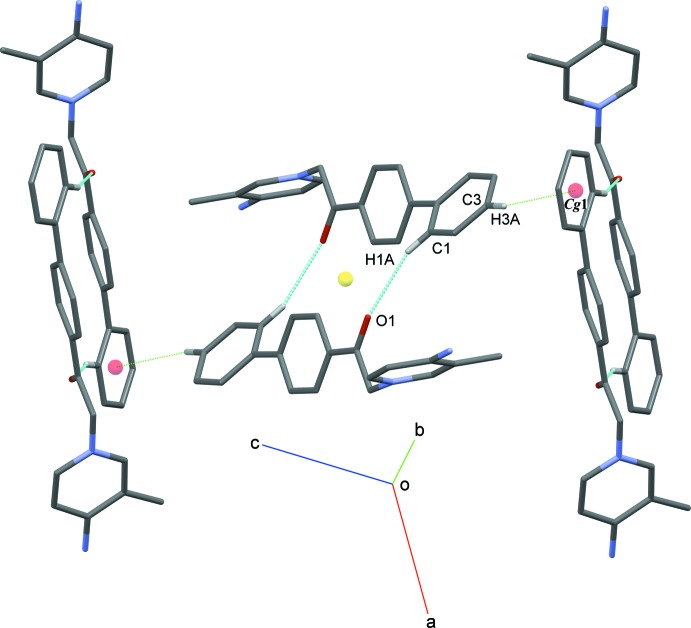
Partial packing diagram of the cation showing the C1—H1*A*⋯O1 hydrogen bonds (blue dashed lines) and C3—H3*A* ⋯π inter­actions (green dashed lines).

In the crystal, the bromide anion is linked to the cation *via* an N2—H*2N2*⋯Br1 hydrogen bond (Table 1 ▸). The bromide anion is surrounded by three other cations with short H⋯Br contracts varying from 2.52 to 2.88 Å (Table 1 ▸). Pairs of C1—H1*A*⋯O1 hydrogen bonds link the pyridiniminium cations into inversion dimers with an 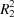
(18) graph-set motif (Table 1 ▸, Fig. 2 ▸). The dimers are stacked in a phen­yl–phenyl T-shaped geometry through C3—H3*A*⋯*Cg*1 inter­actions (*Cg*1 is the centroid of the C1–C6 phenyl ring).

## Hirshfeld surface analysis   

**Figure 3:**
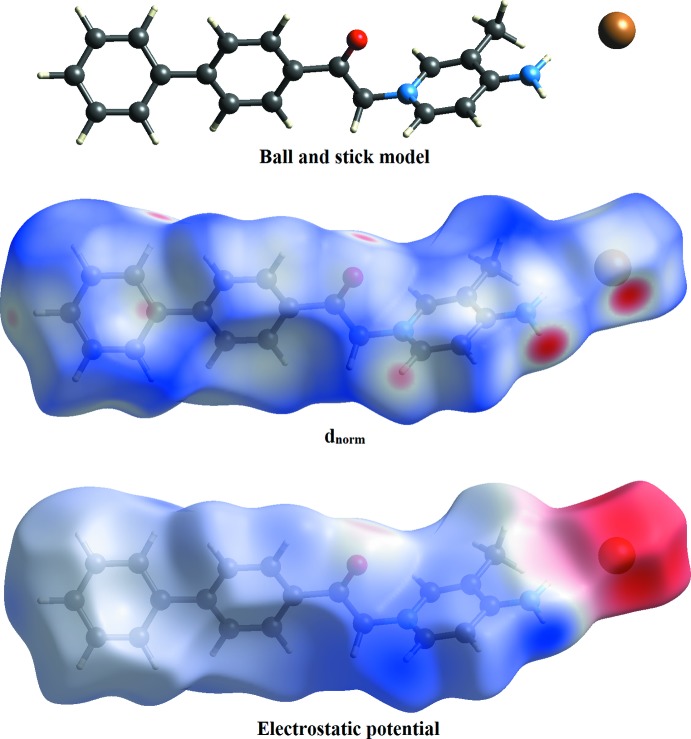
*d*_norm_ and electrostatic potential mapped on Hirshfeld surfaces for visualizing the inter­molecular contacts in the title salt. The ball-and-stick models shown here and in the following figures represent the different orientations corresponding to the Hirshfeld surfaces and their electrostatic potentials.

The Hirshfeld surface analysis (Spackman & Jayatilaka, 2009 ▸) of the title salt was generated by *CrystalExplorer3.1* (Wolff *et al.*, 2012 ▸), and comprised *d*_norm_ surface plots, electrostatic potentials and 2D fingerprint plots (Spackman & McKinnon, 2002 ▸). The ball-and-stick model, *d*_norm_ surface plots and electrostatic potentials of the title salt are shown in Fig. 3 ▸. Those plots were generated to qu­antify and visualize the inter­molecular inter­actions and to explain the observed crystal packing. The dark-red spots on the *d*_norm_ surface arise as a result of short inter­atomic contacts, while the other weak inter­molecular inter­actions appear as light-red spots. Furthermore, negative electrostatic potential (red regions) in the electrostatic potential map indicates hydrogen-acceptor potential, whereas the hydrogen donors are represented by positive electrostatic potential (blue regions) (Spackman *et al.*, 2008 ▸).

**Figure 4:**
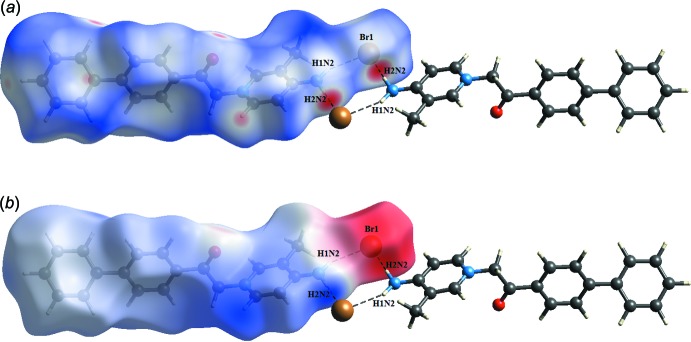
Visualization of N—H⋯O hydrogen bond inter­actions through the (*a*) *d*_norm_ and (*b*) electrostatic potential maps. Hydrogen bonds are represented by dashed lines.

**Figure 5:**
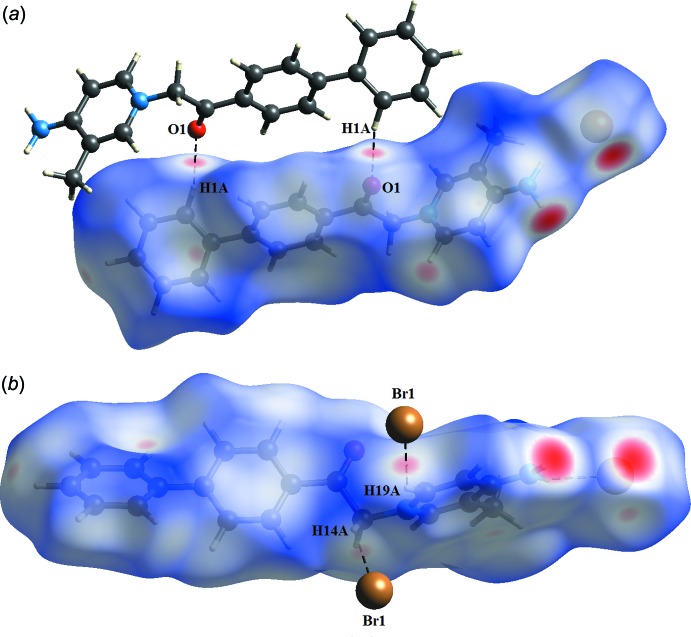
Visualization of (*a*) C—H⋯O hydrogen bonds and (*b*) C—H⋯Br inter­actions through the *d*_norm_ maps. Hydrogen bonds are represented by dashed lines.

**Figure 6:**
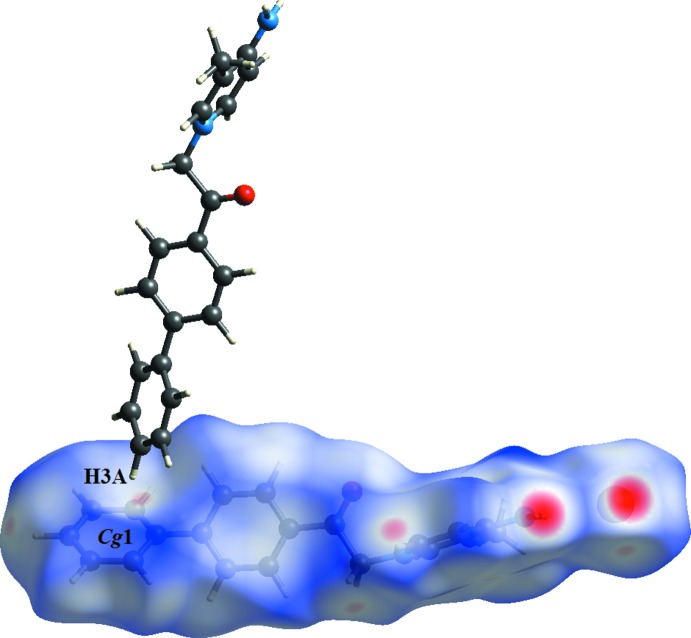
Visualization of C—H⋯π inter­actions through the *d*_norm_ maps.

The *d*_norm_ surface of the title salt shows a dark-red spot on the N–H hydrogen atom and on the bromide atom, which is the result of the strong N2—H*1N2*⋯Br1 and N2—H*2N2*⋯Br1 hydrogen bonds present in the structure (Fig. 4 ▸ *a*). These observations are further confirmed by the respective electrostatic potential maps, where the atoms involved in strong hydrogen bonds are seen as dark-blue and dark-red regions (Fig. 4 ▸ *b*). Beside those two short inter­molecular contacts, the C—H⋯O and C—H⋯Br inter­actions are shown as light-red spots on the *d*_norm_ surface (Fig. 5 ▸). Finally, the C—H⋯π inter­action is shown as a light-red spot on the *d*_norm_ surface (Fig. 6 ▸).

**Figure 7:**
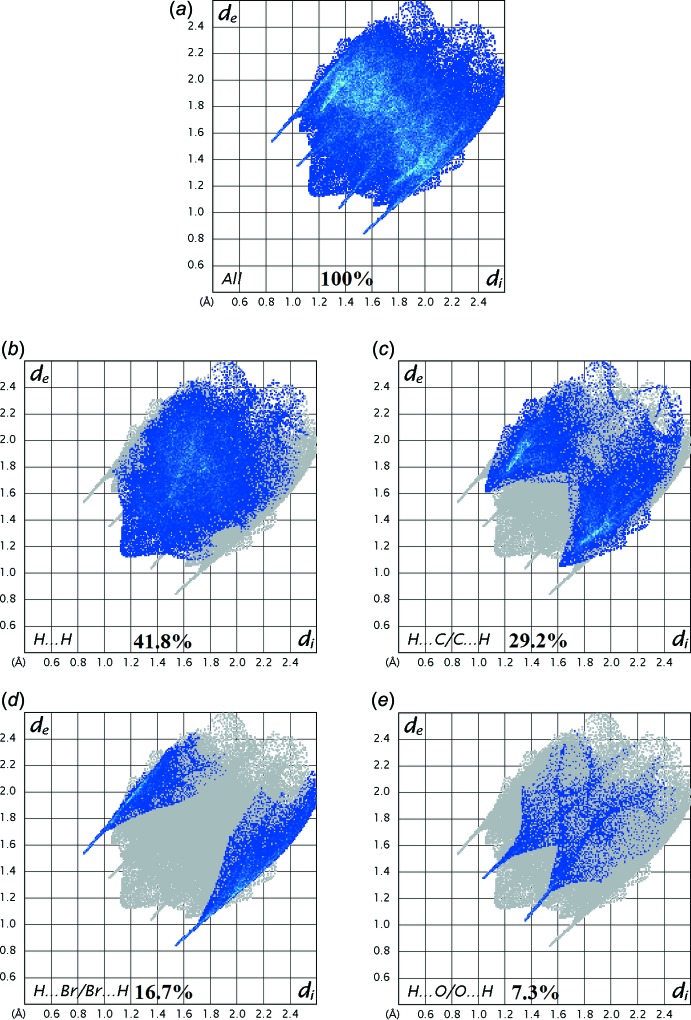
Two-dimensional fingerprint plots of the title salt showing the different percentage contributions for the various types of inter­actions.

A qu­anti­tative analysis of the inter­molecular inter­actions can be made by studying the fingerprint plots (FP). The FP is shown with characteristic pseudo-symmetry wings in the *d*_e_ and *d*_i_ diagonal axes represent the overall two-dimensional FP and those delineated into H⋯H, H⋯C/C⋯H, H⋯Br/Br⋯H and H⋯O/O⋯H contacts, respectively (Fig. 7 ▸). The most significant inter­molecular inter­actions are the H⋯H inter­action (41.8%), which appear at the central region of the FP with *d*_e_ = *d*_i_ ≃ 2.2 Å (Fig. 7 ▸ *b*). The reciprocal H⋯C/C⋯H inter­actions appear as two symmetrical broad wings with *d*_e_ + *d*_i_ ≃ 2.7 Å and contribute 29.2% to the Hirshfeld surface (Fig. 7 ▸ *c*). The reciprocal H⋯Br/Br⋯H and H⋯O/O⋯H inter­actions with 16.7% and 7.3% contributions are present as sharp symmetrical spikes at diagonal axes *d*_e_ + *d*_i_ ≃ 2.3 and 2.4 Å, respectively (Fig. 7 ▸ *d*–*e*). The percentage contributions for other inter­molecular contacts are less than 5% in the Hirshfeld surface mapping.

## Synthesis and crystallization   

**Figure 8:**
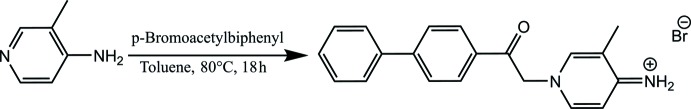
Synthesis of the title compound.

The synthesis of the title compound is illustrated in Fig. 8 ▸. A mixture of 1-([1,1′-biphen­yl]-4-yl)-2-bromo­ethan-1-one (2.75 g, 10 mmol) and 3-methyl­pyridin-4-amine (0.11 g, 1mmol) was dissolved in 10 ml of toluene at room temperature, followed by stirring at 358 K for 18 h. The completion of the reaction was marked by the amount of the separated solid from the initially clear and homogenous mixture of the starting materials. The solid was filtered from the unreacted starting materials and solvent, and subsequently washed with ethyl acetate. The final pyridiniminium salt was obtained after the solid was dried under reduced pressure to remove all volatile organic compounds (Said *et al.*, 2017 ▸). Plate-like yellow crystals were obtained by slow evaporation of a solution in acetone.

## Refinement   

Crystal data, data collection and structure refinement details are summarized in Table 2 ▸. C-bound H atoms were positioned geometrically [C—H = 0.95–0.99 Å] and refined using a riding model with *U*_iso_(H) = 1.2 or 1.5*U*_eq_(C). All N-bound H atoms were located from a difference-Fourier map and freely refined.

## Supplementary Material

Crystal structure: contains datablock(s) global, I. DOI: 10.1107/S2056989018006217/xu5923sup1.cif

Structure factors: contains datablock(s) I. DOI: 10.1107/S2056989018006217/xu5923Isup2.hkl

Supporting information file. DOI: 10.1107/S2056989018006217/xu5923Isup3.cml

CCDC reference: 1839059

Additional supporting information:  crystallographic information; 3D view; checkCIF report

## Figures and Tables

**Table 1:** Hydrogen-bond geometry (Å, °) *Cg*1 is the centroid of the C1–C6 ring.

*D*—H⋯*A*	*D*—H	H⋯*A*	*D*⋯*A*	*D*—H⋯*A*
N2—H1*N*2⋯Br1	0.83 (2)	2.61 (2)	3.3514 (17)	150 (2)
N2—H2*N*2⋯Br1^i^	0.86 (2)	2.52 (2)	3.3763 (17)	174 (2)
C1—H1*A*⋯O1^ii^	0.95	2.52	3.431 (2)	162
C14—H14*A*⋯Br1^iii^	0.99	2.88	3.710 (2)	141
C19—H19*A*⋯Br1^iv^	0.95	2.82	3.5415 (18)	134
C3—H3*A*⋯*Cg*1^v^	0.95	2.81	3.6963 (19)	155

Symmetry codes: (i) 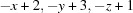; (ii) 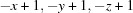; (iii) 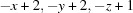; (iv) 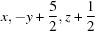; (v) 
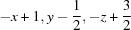
.

**Table 2:** Experimental details

Crystal data	
Chemical formula	C_20_H_19_N_2_O^+^·Br^−^
*M*_r_	383.28
Crystal system, space group	Monoclinic, *P*2_1_/*c*
Temperature (K)	100
*a*, *b*, *c* (Å)	15.3991 (10), 7.9078 (5), 15.7645 (10)
β (°)	113.037 (1)
*V* (Å^3^)	1766.6 (2)
*Z*	4
Radiation type	Mo *K*α
μ (mm^−1^)	2.34
Crystal size (mm)	0.27 × 0.11 × 0.08

Data collection	
Diffractometer	Bruker APEXII DUO CCD area-detector
Absorption correction	Multi-scan (*SADABS*; Bruker, 2012 ▸)
*T*_min_, *T*_max_	0.499, 0.574
No. of measured, independent and observed [*I* > 2σ(*I*)] reflections	28949, 4731, 3913
*R*_int_	0.040
(sin θ/λ)_max_ (Å^−1^)	0.685

Refinement	
*R*[*F* ^2^ > 2σ(*F* ^2^)], *wR*(*F* ^2^), *S*	0.030, 0.075, 1.03
No. of reflections	4731
No. of parameters	226
H-atom treatment	H atoms treated by a mixture of independent and constrained refinement
Δρ_max_, Δρ_min_ (e Å^−3^)	0.70, −0.23

Computer programs: *APEX2* and *SAINT* (Bruker, 2012 ▸), *SHELXS97* (Sheldrick, 2008 ▸), *SHELXL2013* (Sheldrick, 2015 ▸), *Mercury* (Macrae *et al.*, 2006 ▸) and *PLATON* (Spek, 2009 ▸).

## supplementary crystallographic information

### Crystal data

**Table:**

C_20_H_19_N_2_O^+^·Br^−^	*F*(000) = 784
*M**_r_* = 383.28	*D*_x_ = 1.441 Mg m^−^^3^
Monoclinic, *P*2_1_/*c*	Mo *K*α radiation, λ = 0.71073 Å
*a* = 15.3991 (10) Å	Cell parameters from 7970 reflections
*b* = 7.9078 (5) Å	θ = 2.6–28.7°
*c* = 15.7645 (10) Å	µ = 2.34 mm^−^^1^
β = 113.037 (1)°	*T* = 100 K
*V* = 1766.6 (2) Å^3^	Plate, yellow
*Z* = 4	0.27 × 0.11 × 0.08 mm

### Data collection

**Table:**

Bruker APEXII DUO CCD area-detector diffractometer	4731 independent reflections
Radiation source: fine-focus sealed tube	3913 reflections with *I* > 2σ(*I*)
Graphite monochromator	*R*_int_ = 0.040
φ and ω scans	θ_max_ = 29.2°, θ_min_ = 2.6°
Absorption correction: multi-scan (SADABS; Bruker, 2012)	*h* = −20→21
*T*_min_ = 0.499, *T*_max_ = 0.574	*k* = −10→10
28949 measured reflections	*l* = −21→21

### Refinement

**Table:**

Refinement on *F*^2^	0 restraints
Least-squares matrix: full	Hydrogen site location: inferred from neighbouring sites
*R*[*F*^2^ > 2σ(*F*^2^)] = 0.030	H atoms treated by a mixture of independent and constrained refinement
*wR*(*F*^2^) = 0.075	*w* = 1/[σ^2^(*F*_o_^2^) + (0.0375*P*)^2^ + 0.8608*P*] where *P* = (*F*_o_^2^ + 2*F*_c_^2^)/3
*S* = 1.03	(Δ/σ)_max_ = 0.001
4731 reflections	Δρ_max_ = 0.70 e Å^−^^3^
226 parameters	Δρ_min_ = −0.23 e Å^−^^3^

### Special details

**Table:**

Experimental. The following wavelength and cell were deduced by SADABS from the direction cosines etc. They are given here for emergency use only: CELL 0.71093 15.443 7.924 15.800 89.974 113.042 90.030
Geometry. All esds (except the esd in the dihedral angle between two l.s. planes) are estimated using the full covariance matrix. The cell esds are taken into account individually in the estimation of esds in distances, angles and torsion angles; correlations between esds in cell parameters are only used when they are defined by crystal symmetry. An approximate (isotropic) treatment of cell esds is used for estimating esds involving l.s. planes.

### Fractional atomic coordinates and isotropic or equivalent isotropic displacement parameters (Å^2^)

**Table:**

	*x*	*y*	*z*	*U*_iso_*/*U*_eq_	
Br1	0.92732 (2)	1.53732 (2)	0.32391 (2)	0.02112 (6)	
N1	0.85797 (10)	0.77203 (18)	0.52316 (10)	0.0165 (3)	
N2	0.92877 (11)	1.25846 (19)	0.48455 (12)	0.0191 (3)	
H1N2	0.9168 (16)	1.296 (3)	0.4320 (17)	0.028 (6)*	
H2N2	0.9689 (16)	1.310 (3)	0.5315 (16)	0.026 (6)*	
O1	0.68941 (9)	0.74507 (17)	0.53143 (11)	0.0288 (3)	
C1	0.48304 (13)	−0.0147 (2)	0.60543 (12)	0.0190 (3)	
H1A	0.4442	0.0614	0.5597	0.023*	
C2	0.44287 (13)	−0.1546 (2)	0.62926 (12)	0.0217 (4)	
H2A	0.3769	−0.1739	0.5993	0.026*	
C3	0.49803 (13)	−0.2662 (2)	0.69624 (13)	0.0232 (4)	
H3A	0.4703	−0.3613	0.7127	0.028*	
C4	0.59455 (14)	−0.2375 (2)	0.73918 (13)	0.0241 (4)	
H4A	0.6328	−0.3130	0.7856	0.029*	
C5	0.63554 (13)	−0.0997 (2)	0.71483 (13)	0.0209 (4)	
H5A	0.7018	−0.0830	0.7436	0.025*	
C6	0.57967 (12)	0.0150 (2)	0.64790 (12)	0.0166 (3)	
C7	0.62172 (12)	0.1671 (2)	0.62368 (11)	0.0160 (3)	
C8	0.71004 (12)	0.1613 (2)	0.61812 (12)	0.0177 (3)	
H8A	0.7449	0.0587	0.6312	0.021*	
C9	0.74715 (12)	0.3039 (2)	0.59364 (12)	0.0176 (3)	
H9A	0.8072	0.2981	0.5900	0.021*	
C10	0.69697 (12)	0.4563 (2)	0.57416 (12)	0.0159 (3)	
C11	0.60974 (12)	0.4633 (2)	0.58173 (12)	0.0173 (3)	
H11A	0.5756	0.5667	0.5703	0.021*	
C12	0.57262 (12)	0.3209 (2)	0.60576 (12)	0.0181 (3)	
H12A	0.5129	0.3273	0.6102	0.022*	
C13	0.73288 (12)	0.6126 (2)	0.54717 (12)	0.0174 (3)	
C14	0.82786 (12)	0.6034 (2)	0.53803 (13)	0.0187 (3)	
H14A	0.8757	0.5539	0.5948	0.022*	
H14B	0.8224	0.5293	0.4856	0.022*	
C15	0.83058 (12)	0.8351 (2)	0.43666 (12)	0.0178 (3)	
H15A	0.7958	0.7642	0.3861	0.021*	
C16	0.85084 (12)	0.9961 (2)	0.41921 (12)	0.0181 (3)	
C17	0.90417 (11)	1.1006 (2)	0.49601 (12)	0.0160 (3)	
C18	0.92986 (12)	1.0325 (2)	0.58534 (12)	0.0168 (3)	
H18A	0.9638	1.0999	0.6378	0.020*	
C19	0.90626 (12)	0.8711 (2)	0.59677 (12)	0.0175 (3)	
H19A	0.9239	0.8270	0.6573	0.021*	
C20	0.81947 (15)	1.0614 (3)	0.32252 (13)	0.0268 (4)	
H20A	0.7890	0.9700	0.2790	0.040*	
H20B	0.8743	1.1026	0.3118	0.040*	
H20C	0.7745	1.1541	0.3135	0.040*	

### Atomic displacement parameters (Å^2^)

**Table:**

	*U*^11^	*U*^22^	*U*^33^	*U*^12^	*U*^13^	*U*^23^
Br1	0.02487 (10)	0.02019 (10)	0.01683 (9)	−0.00208 (7)	0.00658 (7)	0.00115 (7)
N1	0.0153 (7)	0.0152 (7)	0.0203 (7)	−0.0005 (5)	0.0083 (6)	−0.0004 (6)
N2	0.0222 (8)	0.0166 (7)	0.0195 (8)	−0.0018 (6)	0.0091 (6)	0.0010 (6)
O1	0.0230 (7)	0.0184 (6)	0.0487 (9)	0.0050 (5)	0.0179 (6)	0.0081 (6)
C1	0.0190 (8)	0.0230 (9)	0.0155 (8)	−0.0001 (7)	0.0072 (7)	0.0000 (7)
C2	0.0197 (9)	0.0271 (9)	0.0200 (9)	−0.0050 (7)	0.0097 (7)	−0.0031 (7)
C3	0.0284 (10)	0.0218 (9)	0.0233 (9)	−0.0050 (7)	0.0144 (8)	0.0009 (7)
C4	0.0272 (10)	0.0219 (9)	0.0245 (9)	0.0033 (7)	0.0116 (8)	0.0063 (7)
C5	0.0197 (8)	0.0205 (8)	0.0230 (9)	0.0004 (7)	0.0090 (7)	0.0026 (7)
C6	0.0193 (8)	0.0172 (8)	0.0155 (8)	−0.0003 (6)	0.0093 (7)	−0.0008 (6)
C7	0.0165 (8)	0.0174 (8)	0.0134 (8)	−0.0011 (6)	0.0052 (6)	0.0001 (6)
C8	0.0173 (8)	0.0158 (8)	0.0201 (8)	0.0011 (6)	0.0073 (7)	0.0006 (6)
C9	0.0163 (8)	0.0178 (8)	0.0204 (9)	0.0007 (6)	0.0089 (7)	−0.0007 (7)
C10	0.0160 (8)	0.0165 (8)	0.0145 (8)	−0.0005 (6)	0.0052 (6)	−0.0005 (6)
C11	0.0161 (8)	0.0164 (8)	0.0181 (8)	0.0030 (6)	0.0053 (6)	0.0011 (7)
C12	0.0147 (8)	0.0203 (8)	0.0198 (8)	0.0020 (6)	0.0072 (7)	0.0008 (7)
C13	0.0151 (8)	0.0162 (8)	0.0198 (8)	0.0005 (6)	0.0054 (7)	0.0011 (7)
C14	0.0189 (8)	0.0136 (7)	0.0248 (9)	0.0001 (6)	0.0096 (7)	0.0012 (7)
C15	0.0166 (8)	0.0196 (8)	0.0164 (8)	−0.0010 (6)	0.0056 (7)	−0.0014 (6)
C16	0.0145 (8)	0.0225 (9)	0.0171 (8)	0.0010 (6)	0.0059 (7)	0.0009 (6)
C17	0.0141 (8)	0.0163 (8)	0.0199 (8)	0.0019 (6)	0.0092 (7)	−0.0007 (7)
C18	0.0181 (8)	0.0183 (8)	0.0152 (8)	−0.0007 (7)	0.0078 (6)	−0.0023 (7)
C19	0.0179 (8)	0.0189 (8)	0.0178 (8)	0.0024 (6)	0.0093 (7)	0.0016 (7)
C20	0.0282 (10)	0.0311 (10)	0.0169 (9)	−0.0046 (8)	0.0044 (7)	0.0049 (8)

### Geometric parameters (Å, º)

**Table:**

N1—C15	1.355 (2)	C8—H8A	0.9500
N1—C19	1.356 (2)	C9—C10	1.400 (2)
N1—C14	1.460 (2)	C9—H9A	0.9500
N2—C17	1.337 (2)	C10—C11	1.395 (2)
N2—H1N2	0.83 (2)	C10—C13	1.483 (2)
N2—H2N2	0.86 (2)	C11—C12	1.381 (2)
O1—C13	1.215 (2)	C11—H11A	0.9500
C1—C2	1.389 (3)	C12—H12A	0.9500
C1—C6	1.392 (2)	C13—C14	1.526 (2)
C1—H1A	0.9500	C14—H14A	0.9900
C2—C3	1.383 (3)	C14—H14B	0.9900
C2—H2A	0.9500	C15—C16	1.365 (2)
C3—C4	1.390 (3)	C15—H15A	0.9500
C3—H3A	0.9500	C16—C17	1.430 (2)
C4—C5	1.387 (3)	C16—C20	1.499 (3)
C4—H4A	0.9500	C17—C18	1.412 (2)
C5—C6	1.402 (2)	C18—C19	1.358 (2)
C5—H5A	0.9500	C18—H18A	0.9500
C6—C7	1.485 (2)	C19—H19A	0.9500
C7—C8	1.397 (2)	C20—H20A	0.9800
C7—C12	1.401 (2)	C20—H20B	0.9800
C8—C9	1.385 (2)	C20—H20C	0.9800
			
C15—N1—C19	119.94 (15)	C12—C11—C10	120.46 (16)
C15—N1—C14	120.28 (15)	C12—C11—H11A	119.8
C19—N1—C14	119.51 (15)	C10—C11—H11A	119.8
C17—N2—H1N2	120.3 (16)	C11—C12—C7	120.95 (16)
C17—N2—H2N2	118.3 (15)	C11—C12—H12A	119.5
H1N2—N2—H2N2	120 (2)	C7—C12—H12A	119.5
C2—C1—C6	120.64 (17)	O1—C13—C10	122.65 (16)
C2—C1—H1A	119.7	O1—C13—C14	119.56 (16)
C6—C1—H1A	119.7	C10—C13—C14	117.78 (15)
C3—C2—C1	120.60 (17)	N1—C14—C13	110.35 (14)
C3—C2—H2A	119.7	N1—C14—H14A	109.6
C1—C2—H2A	119.7	C13—C14—H14A	109.6
C2—C3—C4	119.21 (17)	N1—C14—H14B	109.6
C2—C3—H3A	120.4	C13—C14—H14B	109.6
C4—C3—H3A	120.4	H14A—C14—H14B	108.1
C5—C4—C3	120.62 (17)	N1—C15—C16	122.72 (16)
C5—C4—H4A	119.7	N1—C15—H15A	118.6
C3—C4—H4A	119.7	C16—C15—H15A	118.6
C4—C5—C6	120.33 (17)	C15—C16—C17	118.01 (16)
C4—C5—H5A	119.8	C15—C16—C20	121.21 (17)
C6—C5—H5A	119.8	C17—C16—C20	120.78 (16)
C1—C6—C5	118.57 (16)	N2—C17—C18	120.51 (16)
C1—C6—C7	120.31 (16)	N2—C17—C16	121.62 (16)
C5—C6—C7	121.11 (16)	C18—C17—C16	117.87 (16)
C8—C7—C12	118.53 (16)	C19—C18—C17	120.40 (16)
C8—C7—C6	121.51 (15)	C19—C18—H18A	119.8
C12—C7—C6	119.96 (15)	C17—C18—H18A	119.8
C9—C8—C7	120.55 (16)	N1—C19—C18	121.01 (16)
C9—C8—H8A	119.7	N1—C19—H19A	119.5
C7—C8—H8A	119.7	C18—C19—H19A	119.5
C8—C9—C10	120.64 (16)	C16—C20—H20A	109.5
C8—C9—H9A	119.7	C16—C20—H20B	109.5
C10—C9—H9A	119.7	H20A—C20—H20B	109.5
C11—C10—C9	118.85 (16)	C16—C20—H20C	109.5
C11—C10—C13	118.12 (15)	H20A—C20—H20C	109.5
C9—C10—C13	123.02 (16)	H20B—C20—H20C	109.5
			
C6—C1—C2—C3	0.6 (3)	C11—C10—C13—O1	−0.1 (3)
C1—C2—C3—C4	−0.5 (3)	C9—C10—C13—O1	−179.10 (18)
C2—C3—C4—C5	−0.6 (3)	C11—C10—C13—C14	−179.35 (16)
C3—C4—C5—C6	1.5 (3)	C9—C10—C13—C14	1.7 (2)
C2—C1—C6—C5	0.3 (3)	C15—N1—C14—C13	−87.86 (19)
C2—C1—C6—C7	−178.51 (16)	C19—N1—C14—C13	86.16 (18)
C4—C5—C6—C1	−1.4 (3)	O1—C13—C14—N1	8.2 (2)
C4—C5—C6—C7	177.47 (17)	C10—C13—C14—N1	−172.54 (14)
C1—C6—C7—C8	−142.49 (18)	C19—N1—C15—C16	1.1 (3)
C5—C6—C7—C8	38.7 (3)	C14—N1—C15—C16	175.11 (16)
C1—C6—C7—C12	37.5 (2)	N1—C15—C16—C17	0.7 (3)
C5—C6—C7—C12	−141.33 (18)	N1—C15—C16—C20	179.90 (17)
C12—C7—C8—C9	−1.2 (3)	C15—C16—C17—N2	178.41 (16)
C6—C7—C8—C9	178.73 (16)	C20—C16—C17—N2	−0.8 (3)
C7—C8—C9—C10	0.1 (3)	C15—C16—C17—C18	−2.1 (2)
C8—C9—C10—C11	1.4 (3)	C20—C16—C17—C18	178.76 (17)
C8—C9—C10—C13	−179.68 (16)	N2—C17—C18—C19	−178.81 (16)
C9—C10—C11—C12	−1.6 (3)	C16—C17—C18—C19	1.7 (2)
C13—C10—C11—C12	179.36 (16)	C15—N1—C19—C18	−1.6 (2)
C10—C11—C12—C7	0.5 (3)	C14—N1—C19—C18	−175.62 (15)
C8—C7—C12—C11	1.0 (3)	C17—C18—C19—N1	0.2 (3)
C6—C7—C12—C11	−179.00 (16)		

### Hydrogen-bond geometry (Å, º)

Cg1 is the centroid of the C1–C6 ring.

**Table:**

*D*—H···*A*	*D*—H	H···*A*	*D*···*A*	*D*—H···*A*
N2—H1*N*2···Br1	0.83 (2)	2.61 (2)	3.3514 (17)	150 (2)
N2—H2*N*2···Br1^i^	0.86 (2)	2.52 (2)	3.3763 (17)	174 (2)
C1—H1*A*···O1^ii^	0.95	2.52	3.431 (2)	162
C14—H14*A*···Br1^iii^	0.99	2.88	3.710 (2)	141
C19—H19*A*···Br1^iv^	0.95	2.82	3.5415 (18)	134
C3—H3*A*···*Cg*1^v^	0.95	2.81	3.6963 (19)	155

Symmetry codes: (i) −*x*+2, −*y*+3, −*z*+1; (ii) −*x*+1, −*y*+1, −*z*+1; (iii) −*x*+2, −*y*+2, −*z*+1; (iv) *x*, −*y*+5/2, *z*+1/2; (v) −*x*+1, *y*−1/2, −*z*+3/2.
